# Targeted Cell Sorting Combined With Single Cell Genomics Captures Low Abundant Microbial Dark Matter With Higher Sensitivity Than Metagenomics

**DOI:** 10.3389/fmicb.2020.01377

**Published:** 2020-07-22

**Authors:** Hang T. Dam, John Vollmers, Morgan S. Sobol, Angela Cabezas, Anne-Kristin Kaster

**Affiliations:** ^1^Institute for Biological Interfaces 5, Karlsruhe Institute of Technology, Eggenstein-Leopoldshafen, Germany; ^2^Leibniz Institute DSMZ, Brunswick, Germany; ^3^Instituto Tecnológico Regional Centro Sur, Universidad Tecnológica, Durazno, Uruguay

**Keywords:** Chloroflexi, covariant metagenome binning, fluorescence-activated single-cell sorting, fluorescent *in situ* hybridization, multiple displacement amplification, wastewater

## Abstract

Rare members of environmental microbial communities are often overlooked and unexplored, primarily due to the lack of techniques capable of acquiring their genomes. Chloroflexi belong to one of the most understudied phyla, even though many of its members are ubiquitous in the environment and some play important roles in biochemical cycles or biotechnological applications. We here used a targeted cell-sorting approach, which enables the selection of specific taxa by fluorescent labeling and is compatible with subsequent single-cell genomics, to enrich for rare Chloroflexi species from a wastewater-treatment plant and obtain their genomes. The combined workflow was able to retrieve a substantially higher number of novel Chloroflexi draft genomes with much greater phylogenetical diversity when compared to a metagenomics approach from the same sample. The method offers an opportunity to access genetic information from rare biosphere members which would have otherwise stayed hidden as microbial dark matter and can therefore serve as an essential complement to cultivation-based, metagenomics, and microbial community-focused research approaches.

## Introduction

**FIGURE 1:**
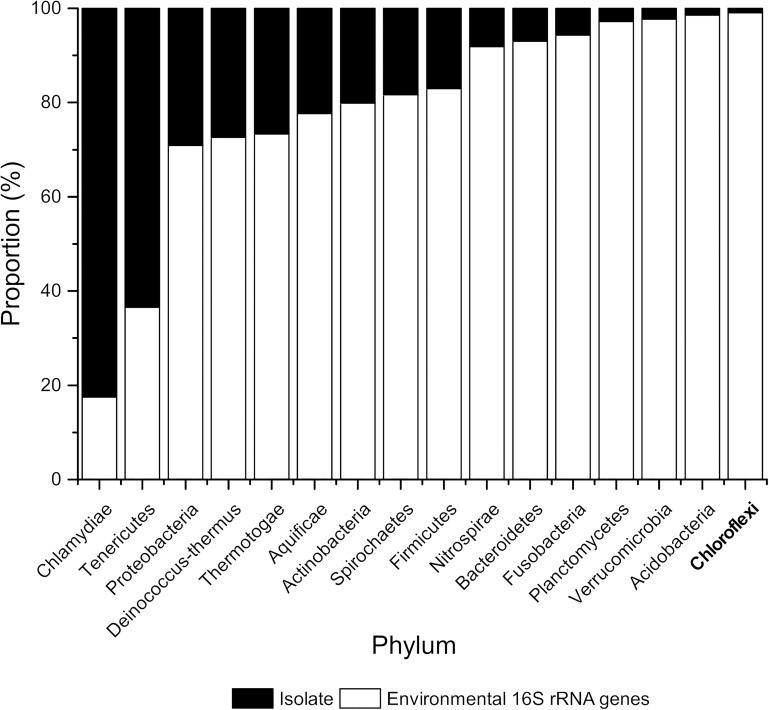
Proportion (%) of isolates and environmental 16S rRNA sequences belonging to selected bacterial phyla based on 16S rRNA sequences. Numbers of 16S rRNA sequences were extracted from the Ribosomal Database Project version 11, updated in September 2016 (Cole et al., 2014).

The vast majority of all microorganisms remain unknown regarding their phylogeny and function, with an estimate of >99% of microbial species not present in axenic cultures (Ward et al., 1992; Torsvik et al., 1996; McCaig et al., 2001). These uncultured microorganisms are often referred to as “microbial dark matter.” Cultivation-independent omics approaches suggest that these organisms are, however, active in their respective habitats, some of them even harboring a potential for biotechnological applications (Hawley et al., 2017; Castelle and Banfield, 2018; Vavourakis et al., 2018). Chloroflexi are a deep-branching lineage within the domain Bacteria. In its current state, the phylum consists of eight classes—*Chloroflexia* (Garrity et al., 2001; Gupta et al., 2013), *Thermomicrobia* (Hugenholtz et al., 1998), *Dehalococcoidia* (Moe et al., 2009; Löffler et al., 2013), *Ktedonobacteria* (Cavaletti et al., 2006; Yabe et al., 2010), *Ardenticatenia* (Kawaichi et al., 2013), *Thermoflexia* (Dodsworth et al., 2014), *Anaerolineae* (Yamada et al., 2006), and *Caldilineae* (Yamada et al., 2007). Metagenomic and 16S rRNA data show that Chloroflexi are ubiquitous throughout the environment; however, only 51 different species have been cultivated so far. Within the SILVA SSU database (version 132, updated in 2017) (Glöckner et al., 2017), there are on the other hand over 9,000 non-redundant sequences deposited and *The Ribosomal Database Project* (version 11, updated in 2016) (Cole et al., 2014) even contains over 22,000 environmental 16S rRNA sequences for Chloroflexi with no corresponding isolate or (draft) genome (Figure 1). Currently, there are 188 draft genomes with more than 90% completeness deposited in the NCBI database as of May 2019^1^ and 546 genomes (>50% complete and <10% contamination) are listed in the Genome Taxonomy Database (GTDB) (Parks et al., 2018). Interestingly, Chloroflexi isolates exhibit a broad diversity of phenotypes and a wide range of metabolic activities (Hug et al., 2013; Islam et al., 2019) and are often found as important members in the environment (Hanada et al., 2002; Taş et al., 2009; Chang et al., 2011; McIlroy et al., 2016; Livingstone et al., 2018). However, as the numbers show they are apparently difficult to cultivate.

Bioinformatic assembly and binning have been the method of choice to obtain (draft) genomes of not-yet-culturable microorganisms like Chloroflexi (Tyson et al., 2004; McIlroy et al., 2017; Andersen et al., 2018) from metagenomes (so-called MAGs, metagenome-assembled genomes). There has been a massive influx of MAGs from novel uncultured microorganisms in the recent past (Hug et al., 2016; Parks et al., 2017; Tully et al., 2018a), and even the recovery of genomes from lower abundant species is now possible due to improvements in sequencing depths and bioinformatics algorithms (Albertsen et al., 2013; Vollmers et al., 2017a, b). As a result, novel phyla, physiological characteristics, and in some cases metabolic pathways could be unraveled at a faster pace than before (Wrighton et al., 2016; Castelle et al., 2017; Sewell et al., 2017). For Chloroflexi, 399 MAGs have so far been deposited in the Genomes Online Database (GOLD) as of May 2019 (Mukherjee et al., 2019). Unfortunately, mobile genetic elements such as plasmids or gene fragments originating from horizontal gene transfer events often cannot be binned accurately from metagenomes. In addition, genome reconstruction for microbes with high genomic heterogeneity levels can result in consensus genomes of closely related taxonomic groups instead of different individual genomes. This is a huge disadvantage when reconstructing genomes from metagenomes, since genomic heterogeneity is a common characteristic of microorganisms to adapt to environments with constant and rapid changes. This holds especially true for microorganisms with low abundance in a habitat, since the quality of genome reconstruction is largely dependent on sequence coverage for assembly as well as coverage covariance-based binning (Vollmers et al., 2017b).

Single-cell genomics (SCG) has emerged as a powerful technique to overcome disadvantages of genome reconstruction from metagenomes (Woyke et al., 2010, 2017; Stepanauskas, 2012; Blainey, 2013). It allows for the physical separation of single cells directly from environmental samples, followed by sequencing and assembly of their individual genomes. An increasing number of single amplified genomes (SAGs) are available from public databases such as GTDB (Parks et al., 2018), NCBI GenBank (Sayers et al., 2020), and/or GOLD (Mukherjee et al., 2019). As of April 2020, 4,907 SAGs have been deposited in GOLD (Mukherjee et al., 2019), of which many are classified as uncultured and potentially novel taxonomic groups (Swan et al., 2011; McLean et al., 2013; Hedlund et al., 2014; Becraft et al., 2016; Landry et al., 2017; León-Zayas et al., 2017). Currently, there are 152 Chloroflexi SAGs deposited in GOLD. However, those SAGs were often recovered from habitats, in which Chloroflexi were naturally enriched (“hot-spots”), such as marine sponges, deep sea sediments, and the dark ocean (Kaster et al., 2014; Wasmund et al., 2014; Landry et al., 2017; Sewell et al., 2017; Bayer et al., 2018).

The conventional SCG workflow to retrieve SAGs requires non-specific staining of microbial populations prior to cell sorting with a fluorescence-activated cell sorter (FACS), whole-genome amplification by multiple displacement amplification (MDA), and screening for SAGs of interest (Rinke et al., 2014). However, this approach is very expensive (Swan et al., 2011) when low-abundant microorganisms are targeted. Like metagenomics, it is therefore not a cost-efficient approach to unravel microbial community members which are not abundant, especially in complex environments. Given the limitations of cultivation-dependent and -independent techniques, minority members of microbial communities are therefore often overlooked and understudied. Nevertheless, they might still play important roles in many biogeochemical processes (e.g., due to high enzyme affinities to certain substrates) or might have biotechnological relevance (Frias-Lopez et al., 2008; Shi et al., 2011; Pratscher et al., 2018). In the recent past, function-driven SCGs has therefore been proposed where single cells are characterized and selected based on a specific functional trait or phenotype of interest, prior to and in conjunction with whole-genome sequencing (Lee et al., 2015; Woyke and Jarett, 2015; Doud and Woyke, 2017). However, function-driven SCGs is difficult to implement on understudied minority members without prior knowledge of their physiology (Doud et al., 2019; Hatzenpichler et al., 2020).

In this study, we used targeted cell sorting to enrich for uncultured Chloroflexi, which account for less than 1% of the total bacterial community in an environmental sample, combined with SCG to obtain their genomes (Supplementary Figure 1). In-solution, fixation-free fluorescence *in situ* hybridization (FISH) was employed as previously described (Podar et al., 2007; Yilmaz et al., 2010; Haroon et al., 2013) to prevent compromising the downstream processes of the SCG workflow, while ensuring high-throughput cell sorting. This combined workflow allowed for the recovery of 19 draft genomes of Chloroflexi species from a Uruguayan winery wastewater treatment plant (WWTP), of which 18 had gone undetected from metagenomics binning of the same environment, even including some members of classes not detected at all in the metagenome.

## Results

### Abundance of the Phylum Chloroflexi in a Uruguayan Winery Wastewater Treatment Plant

**FIGURE 2:**
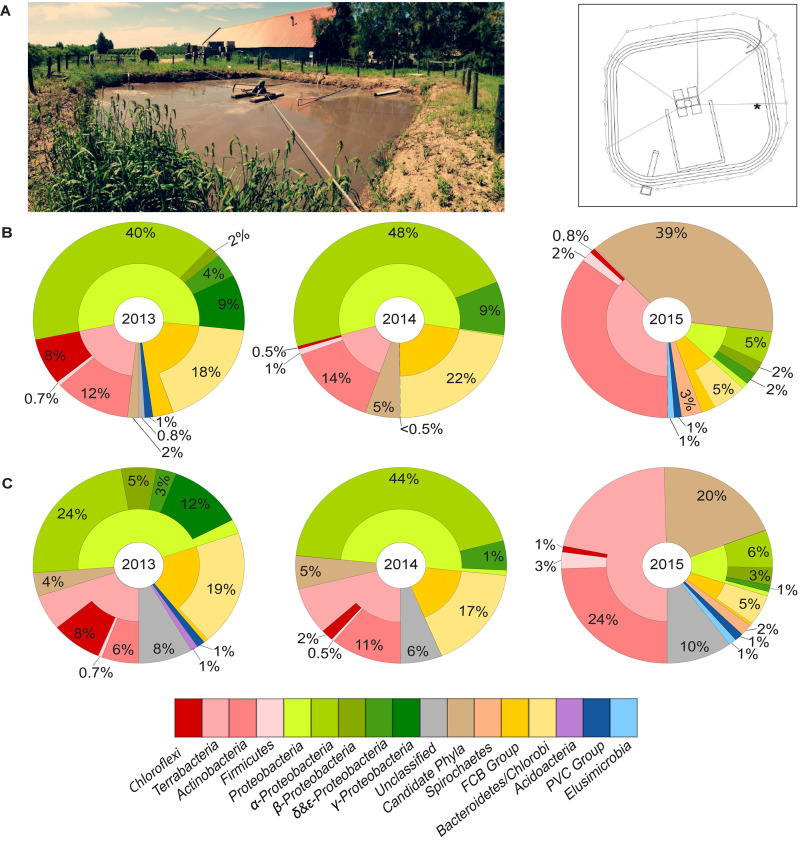
**(A)** Aerated lagoon (LEA) of wastewater treatment plant in Uruguay and its cross-sectional view. The star indicates the exact location where samples were taken. Taxonomic composition of the bacterial community in LEA samples collected in 2013, 2014, and 2015 based on **(B)** 16S rRNA gene sequences and **(C)** single-copy marker genes.

We conducted a survey on the microbial community in an aerated lagoon (Laguna de Ecualizacion y Aireacion, now referred to as LEA), which is part of a WWTP receiving effluent from the Juanicó winery (Canelones, Uruguay) over the course of three years (2013, 2014, and 2015) (Figure 2A). The effluent’s volume and composition varied greatly depending on the winery’s operation. Chemical oxygen demand (COD) fluctuated between 250 and 25,000 mg/L, causing a frequent and massive change in composition of microbial communities, including Chloroflexi. Preliminary surveys using pyrosequencing showed a large fluctuation in abundances of Chloroflexi, with up to 50% of Chloroflexi 16S rRNA gene sequences found in some of the samples (Supplementary Figure 2).

### Draft Genomes of Chloroflexi Using Metagenomics

Microbial communities in three samples from the years 2013, 2014, and 2015 were studied in-depth using metagenomics. DNA libraries using DNA extracted from these samples were sequenced, yielding 11, 10, and 88 million raw read-pairs and 5, 4, and 26 gigabases (Gbp) of sequence information, respectively, deposited at the NCBI short read archive (SRA) under accession numbers SRR10961541–SRR10961543. This resulted in metagenomic assembly sizes of 237, 173, and 802 Mbp, respectively. Based on the read abundance of both 16S rRNA and single-marker genes, Proteobacteria, Bacteroides, and Actinobacteria dominated the microbial communities in samples collected in 2013 and 2014 and were present at different relative abundances at the different time points (Figures 2B,C). Interestingly, bacteria belonging to the candidate phyla radiation predominated the community in the sample collected in 2015, which accounted for 39% of the total 16S rRNA sequences or 20% based on marker gene analysis. The phylum Chloroflexi accounted for a substantially high fraction in the total community of the sample collected in 2013 (8%) but drastically reduced to less than 1% in the sample collected in 2015 based on both 16S rRNA and marker gene analyses (Figures 2B,C). The sample from 2015 (now referred to as LEA2015) was therefore sequenced more deeply and chosen for testing the efficiency of our targeted cell-sorting approach.

**FIGURE 3:**
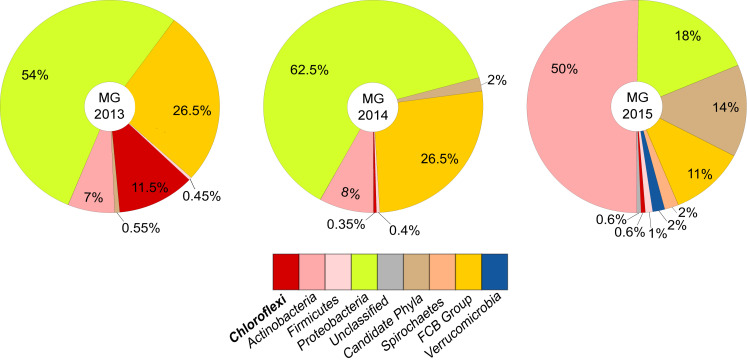
Taxonomic composition and relative abundances of organisms represented by metagenome assembled genomes (MAGs) from WWTP samples over a period of three years. Relative proportions between MAGs were determined based on average contig coverages of each MAG in relation to the overall coverage of binned contigs for each metagenome, thereby correcting for potential differences in genome size and completeness. The fraction of sequence information that could be successfully binned represented 67–73% of the sequence data that could be assembled into contigs > 1 kbp (see also Supplementary Figure 7). The resulting relative abundances between binned taxa largely reflect the corresponding taxon proportions of the community observed *via* marker gene analyses of the complete metagenomes (see also Figure 2).

Binning the sequence data from the metagenome of LEA2015 only retrieved two Chloroflexi MAGs. To determine the possibility of recovering more Chloroflexi in the same WWTP sample, differential coverage binning was performed using all three metagenomes of the LEA samples collected in the 3 years. The three datasets differed in relative abundances of almost all phyla; the most striking differences were relative abundances of Chloroflexi (by a factor of 10) and candidate phyla radiation (by a factor of 19.5) (Figures 2B,C). The binning produced 113 total bins (Supplementary Table 1), of which four where Chloroflexi. These four bins showed high completeness estimates between 50.2 and 93.2% and low contamination levels (<2.5%). Overall, Chloroflexi bins accounted for 11.5, 0.35, and 0.6% of total bins in LEA2013, 2014, and 2015 metagenomes, respectively (Figure 3). The four bins could be classified within three different classes: *Ardenticatenia* (Clx_MAG1 and Clx_MAG4), *Thermomicrobia* (Clx_MAG2), and *Anaerolinea* (Clx_MAG3) (Table 1).

**TABLE 1:** Overview of Chloroflexi co-assembled genomes (CAGs^a^) and single amplified genomes (SAGs) obtained from targeted cell sorting combined with single-cell genomics and metagenome assembled genomes (MAGs) from the LEA2015 sample.

Genome	Compl. (%)	CheckM contamination Statistics* [Cont./SH/Adj-Cont.] (%)	Size (Mbp)	GC (%)	Classification	NCBI accession
**Chloroflexi CAGs and SAGs**						
Clx_CAG1	89.81	8.7/0/**8.7**	6.97	53.4	*Caldilineae*	JAAEJZ000000000
Clx_CAG2	75.71	2.3/0/**2.3**	3.41	62.5	*Anaerolineae*	JAAEKA000000000
Clx_CAG3	28.74	0/0/**0**	1.91	58.9	*Caldilineae*	JAAEKB000000000
Clx_SAG4	55.17	1.72/0/**1.72**	2.12	61.3	*Anaerolineae*	JAAEKC000000000
Clx_SAG5	49.14	0/0/**0**	3.37	52.9	*Caldilineae*	JAAEKE000000000
Clx_SAG6	49.06	1.72/0/**1.72**	1.73	49.1	*Anaerolineae*	JAAEKF000000000
Clx_SAG7	45.05	2.41/75/**0.6**	0.63	36.1	Unclassified	JAAEKG000000000
Clx_SAG8	23.82	0.16/0/**0.16**	0.72	50.6	*Ardenticatenia*	JAAEKH000000000
Clx_SAG9	23.67	0/0/**0**	1.42	63.0	*Caldilineae*	JAAEKI000000000
Clx_SAG10	20.06	0.34/0/**0.34**	1.10	57.6	*Ardenticatenia*	JAAEKJ000000000
Clx_SAG11	19.28	0/0/**0**	0.51	60.6	*Caldilineae*	JAAEKK000000000
Clx_SAG12	18.97	0/0/**0**	0.72	50.6	*Anaerolineae*	JAAEKL000000000
Clx_SAG13	16.85	0/0/**0**	0.88	47.3	*Anaerolineae*	JAAEKN000000000
Clx_SAG14	16.14	1.72/0/**1.72**	0.58	45.4	*Anaerolineae*	JAAEKO000000000
Clx_SAG15	14.42	0.16/0/**0.16**	1.18	61.9	*Ardenticatenia*	JAAEKP000000000
Clx_SAG16	14.33	0/0/**0**	1.72	59.0	Cand. *Thermofonsia*	JAAEKQ000000000
Clx_SAG17	13.95	0/0/**0**	0.87	45.4	*Anaerolineae*	JAAEKR000000000
Clx_SAG18	10.82	0/0/**0**	0.54	60.0	*Chloroflexia*	JAAEKS000000000
Clx_SAG19	5.17	0/0/**0**	0.12	46.6	*Caldilineae*	JAAEKT000000000
**MAGs**						
Clx_MAG1	92.37	5.83/33.33/**3.89**	5.32	61.4	*Ardenticatenia*	JAACJX000000000
Clx_MAG2	67.63	0/0/**0**	1.69	60.5	*Thermomicrobia*	JAACJY000000000
Clx_MAG3	75.24	1.33/50/**0.67**	1.64	55.7	*Anaerolineae*	JAACJZ000000000
Clx_MAG4	48.12	3.45/50/**1.72**	3.09	64.4	*Ardenticatenia*	JAACKA000000000

^*a*^CAGs were co-assembled from multiple SAGs as followed when 16S rRNA gene sequences resulted from screening shared at least 99% similarity and the average nucleotide identity values (ANI) determined by the Pyani package of preliminary bins shared more than 98% over at least 10% genome coverage (component SAGs listed with corresponding CheckM completeness estimations in square brackets): Clx_CAG1 (SAG1 [42%], SAG34 [36%], SAG35 [42%], SAG37 [21%], SAG38 [46%], SAG39 [35%], SAG40 [9%]); Clx_CAG2 (SAG2 [58%], SAG28 [19%], SAG29 [27%], SAG31 [30%]); Clx_CAG3 (SAG3 [12%], SAG27 [16%], SAG32 [11%], SAG33 [10%]). * Completeness and contamination estimations were based on CheckM results (Parks et al., 2015) using universal bacterial-specific marker sets. Corresponding estimations based on Chloroflexi-specific marker sets can be found in Supplementary Table 8. The CheckM “contamination” statistics are given in the form of three values: “Cont.”, original CheckM contamination estimate, based on the number of duplicate markers; “SH”, “strain heterogeneity” indicating the fraction of duplicate markers with almost complete sequence identity not reflecting cross species contamination; “Adj.-Cont.”, “adjusted contamination” giving the fraction of duplicate marker genes with distinct sequence.

### Capturing Rare Chloroflexi in a WWTP Sample Using Targeted SCG

Targeted cell sorting was first validated using a mock culture containing 1% of a known Chloroflexi isolate, *Sphaerobacter thermophilus* (DSM20745), and 99% *Escherichia coli* K12 (DSM498) before applying it to an environmental sample (Supplementary Figure 3). Two previously designed probes (CFX1223 and GSNB941), which target Chloroflexi 16S rRNAs, were used in a ratio of 1:1 to increase hybridization signals (Gich et al., 2001; Björnsson et al., 2002). The probes were first tested on different Chloroflexi classes and were successfully detected under an epifluorescence microscope as well as on the scattergram of the FACS. Under the same hybridization conditions, a culture containing only *E. coli* did not exhibit any fluorescent signals. The FISH-labeled mixture was sorted in two consecutive steps: first an enrichment sort and then a single-cell sort. The sorted population was gated based on its enhanced fluorescent signal compared to the non-labeled mixed culture. The gated population was greatly enriched from 0.9 to 76% after the first sorting step (Supplementary Figure 3). Phylogenetically labeled cells remained intact and exhibited sufficient fluorescent signals for multiple sorts under considerably high pressure during the sorting with the FACS, proving the performance of the technique.

**FIGURE 4:**
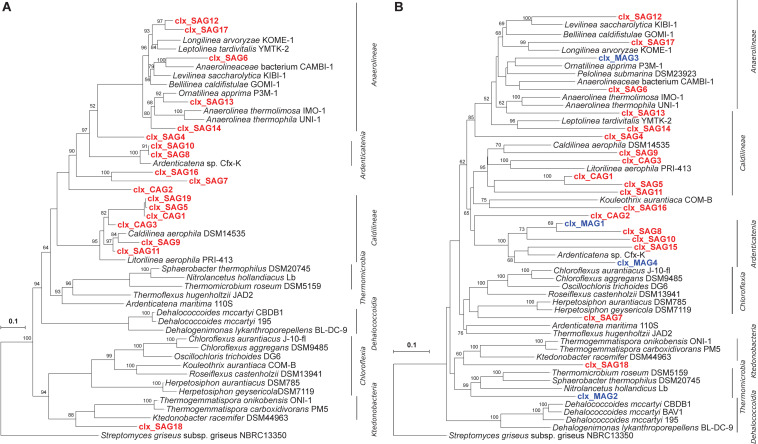
Phylogenetic analysis: **(A)** 16S rRNA genes of Chloroflexi single amplified genomes (SAGs) constructed using the Arb software. Partial 16S rRNA genes of approximately 500 bp were amplified from SAGs; their sequences were aligned using the online SINA platform and imported into Arb. 16S rRNA gene sequences extracted from genomes of Chloroflexi isolates are included as references. **(B)** Distance-based phylogeny on the basis of gene content of Chloroflexi genomes obtained *via* SCG and metagenomics approaches. Classification at the class level is given on the right. *Streptomyces griseus* is used as an outgroup. Nodes with bootstrap values of more than 50 are presented in the tree. Scale bars indicate a 10% sequence divergence.

The low-abundant Chloroflexi cells in WWTP sample LEA2015 were then hybridized using in-solution fixation-free FISH with Chloroflexi-specific probes and sorted on the FACS (Supplementary Figure 4). Whole-genome amplification of the sorted cells using MDA resulted in 1,425 SAGs, yielding 2,000–5,000 ng DNA. Due to the biased nature of the MDA reaction (Lasken, 2009), 505 SAGs showed a positive PCR amplification (35.4% of total SAGs) using a broad eubacterial primer pair targeting the bacterial 16S rRNA gene (Rinke et al., 2014). The majority of the Sanger sequencing chromatograms (>95%) exhibited high-quality signals with minimal background noise similar to those of the isolates, suggesting that the sorted cells were indeed single cells. A phylogenetic analysis of the 16S rRNA sequences revealed that 41 SAGs could be clearly classified as Chloroflexi, some having the same 16S rRNA sequence. These 41 SAGs were considered novel Chloroflexi species because the majority showed less than 94% 16S rRNA sequence identity to any known Chloroflexi isolates and none showed identities of 98% or higher. Based on 16S rRNA phylogeny after screening, 19 SAGs were associated with the class *Caldilineae*, 18 with *Anaerolineae* and three with *Ardenticatenea*. One SAG could not be assigned to any recognized class of Chloroflexi. Within the phylum, it is most closely related to *Thermosporothrix* species within the class *Ktedonobacteria* with 86.5% 16S rRNA gene sequence similarity (Figure 4A). Considering the fact that only 0.8% of Chloroflexi were present in the bacterial community of LEA2015 (Figure 2B), non-specific labeling prior to cell sorting would have statistically resulted in only 4 Chloroflexi SAGs within the identified 505 SAGs, provided that all cells in the sample had the same GC content and lysed equally well. Therefore, our results show that the number of Chloroflexi SAGs which were captured using targeted cell sorting increased by a factor of 10 compared to using non-specific labeling.

### Draft Genomes of Chloroflexi Using Targeted SCG

Initial assemblies of Chloroflexi single cells were created and some later co-assembled. The co-assembly resulted in three Chloroflexi genomes, now referred to as co-assembled genomes—CAGs, with substantially improved genome completeness (i.e., Clx_CAG1 from 7 SAGs, Clx_CAG2 from 4 SAGs, and Clx_CAG3 from 4 SAGs). This resulted in 19 different draft genomes of Chloroflexi species (Table 1). Taxonomic assignment, based on a hierarchical least common ancestor (LCA) contig classification approach, revealed that nine draft genomes belonged to *Anaerolineae*, six to *Caldilineae*, three to *Ardenticatenia*, and one to *Chloroflexia*. Interestingly, two SAGs (Clx_SAG7 and Clx_SAG16) could not be placed in any predefined classes (Table 1) and therefore were considered either as unclassified Chloroflexi or the candidate class *Thermofonsia*. Two SAGs (SAG30 and SAG41) were initially classified as Chloroflexi based on 16S rRNA screening but were later clustered within the phyla Saccharibacteria and Bacteroidetes, respectively, based on their genome content. On the other hand, three single cells identified as candidate phyla based on Sanger sequencing of their 16S rRNA genes were later classified as Chloroflexi and two were shown to contain fragments of 16S rRNA genes from different species after whole-genome sequencing (Supplementary Table 2), indicating that in these cases more than one cell got sorted into the same well. In addition to the phylogeny of the 16S rRNA genes from Chloroflexi CAGs and SAGs, relationships were also inferred on the basis of gene content clustering using Chloroflexi genomes obtained *via* SCG and metagenomics binning approaches which had more than 10% genome completeness (Figure 4B). The targeted cell-sorting approach retrieved almost 10 times more “clean” Chloroflexi genomes than metagenomics binning from the same sample and almost five times more genomes when using covariant binning with three metagenomic samples.

### Potential Metabolic Properties of the Novel Chloroflexi Species

Preliminary functional analysis of the draft genomes obtained *via* targeted cell sorting indicated that the Chloroflexi found in the Uruguayan winery WWTP may exhibit a heterotrophic lifestyle. They are likely to be involved in the transformation and degradation of carbohydrates and aromatic compounds, which are highly enriched in wastewater environments. Genes associated with aromatic compound degradation, such as cytochrome p450-dependent monooxygenase, dioxygenase ferredoxin, and catechol 2,3-dioxygenase, were found in several of the Chloroflexi draft genomes, although no complete pathway could be inferred from the data. Furthermore, four out of six *Caldilineae* draft genomes (Clx_CAG1, Clx_CAG3, Clx_SAG5, and Clx_SAG11) showed indications for genes involved in aerobic respiration: succinate dehydrogenase, NADH-quinone oxidoreductase, cytochrome c oxidase, and an F-type H^+^ transporting ATPase, although not all subunits were found. This is in accordance not only with the aerobic conditions of the habitat sampled in this study but also with the observation of an aerobic lifestyle in *Caldilineae* isolates from hot springs (Sekiguchi et al., 2003; Kale et al., 2013). Interestingly, Clx_SAG11, which clustered within *Caldilineae* and was only retrieved *via* the targeted SCG approach, contained marker genes indicative of carbon fixation *via* the Calvin–Benson–Bassham (CBB) cycle, i.e., ribulose bisphosphate carboxylase (*cbb*) and phosphoribulose kinase (*prk*). The amino acid sequence of the large subunit of ribulose bisphosphate carboxylase (CbbL) had the highest sequence identity with that of a Chloroflexi isolate—*Kouleothrix aurantiacus*. CbbL of Clx_SAG11 also had high sequence identity and clustered with sequences from three other Chloroflexi isolates belonging to the classes *Chloroflexia* and *Thermomicrobia* (Supplementary Figure 5). A gene cluster containing ribulose bisphosphate carboxylase large (*cbbL*) and small (*cbbS*) subunits, a putative regulator gene (*cbbX*), and phosphoribulose kinase (*prk*) was similar to those of gene clusters of bacterial RubisCo type I. Previously, this feature has not been observed in any *Caldilinea* isolate. Notably, CAG1, a novel Chloroflexi species with an estimated size of almost 7 Mbp, harbored 57 putative secondary metabolite synthesis gene clusters, some potentially involved in synthesis of terpene, type III polyketide synthase, non-ribosomal peptide synthase, and bacteriocin (Supplementary Figure 6), making this species a potential candidate for production of biologically active compounds.

## Discussion

### Capturing Rare Microorganisms by Targeted Cell Sorting Combined With SCG

Obtaining genomes of rare microorganisms through culture-independent methods requires either extensive sampling over a long period of time to capture the moment when rare species become dominant or very deep sequencing, if at all possible (Hugenholtz et al., 1998; Köpke et al., 2005). Some species with unique, important, and rate-limiting biological functions (such as nitrification, methane and methanol oxidation, or respiratory dehalogenation) are however permanently found at low abundance in the environment (Griffiths et al., 2004; Dam and Häggblom, 2017; Pratscher et al., 2018). Nevertheless, those rare microorganisms play important ecological roles and contribute to biodiversity and ecological cycles more than previously known (Jousset et al., 2017). Some rare organisms might also serve as a “seed bank” and become dominant when ecological conditions become favorable (Shade et al., 2014; Lynch and Neufeld, 2015; Fuentes et al., 2016).

Attempts to retrieve genomes of Chloroflexi directly from the environment using SCG was so far only possible for environmental samples known as hotspots for the phylum such as the deep ocean, subseafloor sediments, and marine sponges, where Chloroflexi constituted 10–70% of the total communities (Kaster et al., 2014; Wasmund et al., 2014; Fullerton and Moyer, 2016; Landry et al., 2017; Sewell et al., 2017; Bayer et al., 2018). Since the standard cell lysis applied prior to whole-genome amplification is not applicable to all types of cells, the success of the MDA reaction of single cells sorted from environmental samples can range from 10 to 40% (Rinke et al., 2014). In addition, the average percentage of successfully amplified 16S rRNA genes for screening is only 30–40% (Kaster et al., 2014; Rinke et al., 2014; Fullerton and Moyer, 2016) because of the biased MDA reaction (Lasken, 2009). For these reasons, SCG becomes statistically very expensive when recovering genomes of rare species of a habitat. In this study, capturing rare uncultured Chloroflexi species was feasible using targeted cell sorting using a modified fluorescent *in situ* hybridization protocol combined with SCG. This combined methodology offers an innovative approach to access genetic information of minority members of any taxonomic group in order to gain better understanding of their ecological roles and potential biotechnological applications (Podar et al., 2007; Lee et al., 2015).

Usually, fluorescent labeling of bacterial cells requires fixatives such as paraformaldehyde to increase the fluorescent signal by allowing a stronger permeabilization of the cell membrane and penetration of the probe. However, since the process weakens the cell wall it can lead to the lysis of the labeled cells during the sorting. Furthermore, paraformaldehyde compromises the downstream applications for SCG, namely, the amplification of the genomic DNA *via* MDA (Clingenpeel et al., 2014; Doud and Woyke, 2017). We here demonstrated that this in-solution fixation-free FISH protocol allowed phylogenetically labeled cells to remain intact during the sorting process and that their fluorescent signals were sufficiently high for multiple sorts. This was archived by longer hybridization times and higher probe concentrations to overcome the problem of low cell-membrane permeability when no additional fixatives and lysing agents could be used. The success of single-cell genome amplification (an average of 38.6% of sorted cells were successfully amplified) was also well within those reported from studies using conventional SCGs (Swan et al., 2011; Kaster et al., 2014; Rinke et al., 2014).

In general, whole-genome amplification *via* MDA to obtain sufficient quantity of genomic DNA for sequencing remains the major limitation of the SCG pipeline. This method often results in incomplete and uneven genome amplification and is biased against high GC regions of the genome. Therefore, the average completeness of genomes (32%) obtained by SCG in this study is lower than that of MAGs from the same sample (68%); however, the SAGs were well within that of other Chloroflexi SAGs obtained *via* SCG pipelines without specific labeling (Kaster et al., 2014; Fullerton and Moyer, 2016; Landry et al., 2017; Sewell et al., 2017). To overcome the problem of amplification bias, a thermotolerant phi29 DNA polymerase could be used in the future, which was recently shown to result in higher-quality draft genomes of single sorted cells (Stepanauskas et al., 2017) as well as the addition of certain additives that prevent secondary structure formation.

Notably, a relatively high proportion of the screened single cells (14%) were classified as candidate phyla (Shapirobacteria, Moranbacteria, and Nomurabacteria) which were also present at very low abundance in the LEA2015 sample (less than 0.1%). Analysis of their 16S rRNA sequences showed that the Chloroflexi probe CFX1223 had only one mismatch to their sequences (Klindworth et al., 2013). Further examination of the probe GNSB941 also showed that it only had one mismatched nucleotide to approximately 0.2% and 0.3% of the 16S rRNA sequences of Firmicutes and Bacteroidetes, respectively. Since the sample was pretreated with ethanol to generally enhance the efficiency of probe penetration into the cells, it consequently also increased the likelihood for “unspecific” hybridization events. However, further experiments could prove that ethanol pretreatment is not necessary to label Chloroflexi. Omitting the ethanol treatment during the FISH protocol would also increase the completeness of the SAGs as ethanol was shown to significantly reduce genome coverage (Clingenpeel et al., 2014). Also, unspecific labeling may be further reduced by adding additional Chloroflexi-specific probes that target different 16S rRNA regions. Another possibility to increase the percentage of the targeted single cells would be to combine labeling of rare Chloroflexi and negative labeling of dominant but unwanted taxa using taxa-specific probes with different fluorophores. The dominant but unwanted taxa could be recognized and sorted out by the cell sorter in a presort. The co-concurrent labeling strategy could drastically increase the number of wanted microorganisms that are sorted.

### Comparing Metagenomics and Targeted SCG for Retrieving Genomes of Rare Biosphere Members

For the WWTP samples, the fraction of sequencing reads incorporated into contigs larger than 1 kb was 46–70%. This is well within the range of observations by other studies (Howe et al., 2014; Frank et al., 2016; Vollmers et al., 2017b), where the fraction of assembled reads could be as low as 10%—and even then mostly represent contigs shorter than 1 kb. The fraction of reads that actually contributed to any of the obtained MAGs was only 33–47% (Supplementary Figure 7).

SAGs are also affected by fragmented assemblies, predominantly due to the uneven read coverage caused by MDA bias. However, since binning is not required, contigs below 1 kb also contribute to the reconstruction of genomes. MDA bias has been shown to be not entirely random, as secondary structures and high GC contents can cause affected portions of the genome to be systematically underrepresented (Ballantyne et al., 2007; Marine et al., 2014; Sabina and Leamon, 2015). Therefore, extreme sequencing depths may help to increase coverage of underrepresented genome regions and enable a more complete genome assembly. In order to verify this effect, varying sequencing depths were applied for the Chloroflexi SAGs ranging from 2 to 16 million reads per single cell. However, post-assembly mapping data showed that the information gain by extreme sequencing depths, represented by contigs with down to 2 × read coverage, was, in most cases, minimal (Supplementary Figure 8). An average sequencing depth of 5 million reads per genome showed to be sufficient, which is in agreement with the results by the Bigelow Single Cell Genomics Center^2^. The fact that several SAGs already display higher metagenome coverage than several of the MAGs indicates that an increase in metagenome coverage may not necessarily guarantee successful binning (Supplementary Table 7).

Among the four Chloroflexi MAGs obtained *via* differential coverage binning from three LEA metagenomes, the most complete genome (MAG1) was also retrieved *via* targeted cell sorting. MAG1 (related to *Promineofilum breve*, belonging to the class *Ardenticatenia*) shared the same taxonomic assignments, a similar coverage variation profile, and a 99% average nucleotide identity (ANI) over 11–17% genome coverage with Clx_SAG8, Clx_SAG10, and Clx_SAG15. It is therefore likely that these genomes originate from the same *Ardenticatenia* species. Although MAG1 appears more complete than the SAGs, it seems that unlike the three SAGs the genome does not contain genomic islands, such as vectors of horizontal gene transfer. This is a known drawback of metagenomic binning (Dick et al., 2009) and could be directly assessed through comparisons, where we found several cases of genomic islands present in the SAGs but not in the MAG (Supplementary Table 3). Examples include a 54.82-kb putative phage contig in Clx_SAG8 and several instances of transposon-associated genes of various putative functions in all three corresponding SAGs. Another known drawback of metagenomes is the possible co-assembly or co-assignment of multiple similar strain variants into a single-consensus genome. This may be reflected by the apparent higher contamination estimates for the MAG compared to the corresponding SAGs.

MAG2, clustering within the class *Thermomicrobia*, was the only genome not captured using the targeted SCG approach. This MAG was classified in the family *Sphaerobacteriaceae*, notoriously known for rigor cell wall structure (Pati et al., 2010). The standard cell lysis condition used in our workflow was likely not sufficient to capture this species, illustrating the necessity of optimizing the lysis step in order to further increase the overall sensitivity of SCG approaches.

Although genome completeness of Chloroflexi SAGs was lower than that of Chloroflexi MAGs, the novel targeted sorting approach showed a higher sensitivity for low-abundant microbial dark matter by capturing much more phylogenetically diverse representatives than metagenomics binning. For example, the *Caldilineae* and *Chloroflexia* classes were not recovered at all using the metagenomic binning approach (Table 1 and Supplementary Tables 1, 4). In addition, Candidatus *Thermofonsia* (Clx-SAG16) and an unclassified Chloroflexi (Clx_SAG7) were also not captured *via* the metagenomics approach at the applied sequencing depth. Based on NCBI taxonomy, Clx_SAG7 could not be reliably assigned to a higher taxon level beyond the phylum Chloroflexi, indicating an association with a potential novel class clustering basally to the phylum Chloroflexi, an interpretation that is supported by 16S rRNA phylogeny and gene-content clustering (Figure 4). In addition, a novel metabolic property of a *Caldilineae* (Clx_SAG11), namely, fixing carbon dioxide *via* the CBB cycle, could be unraveled.

### Ecological Significance of Chloroflexi

In this study, matching partial genomes related to Candidatus *Promineofilum breve*, belonging to the class *Ardenticatenia* (McIlroy et al., 2016), were recovered by both metagenomics binning (MAG1) and the targeted SCG approach (Clx_SAG8, Clx_SAG10, and Clx_SAG15). Previously known as Eikelboom phylotype 0092, this species was frequently found in activated sludge in WWTPs at a relatively high abundance (Speirs et al., 2009; McIlroy et al., 2016). The bacterium was present at a relatively high abundance in the LEA sample collected in 2013, constituting 88% of all Chloroflexi and 11% of all bacteria. Due to its filamentous morphology, it might play vital roles in floc formation and sludge settling and might be associated with bulking episodes in WWTPs. It has a versatile mode of metabolism, including the ability to use oxygen, nitrite, and nitrous oxide for respiration as well as ferment various carbohydrates. As a result, it grows to a great extent when nutrients become abundant and contributes to the transformation of organic matter in the treatment process of winery wastewater (McIlroy et al., 2016).

The other genomes of Chloroflexi species that were only recovered *via* our SCG approach, in contrast, were present at a very low abundance over the sampling times. However, their role in this habitat cannot be overlooked. They shared the ability to metabolize a variety of carbohydrates, as inferred from the genome annotation, and might equally contribute to organic material degradation as more abundant members. This functional redundancy among these organoheterotrophic Chloroflexi species might also help maintain a balanced and “healthy” wastewater treatment system by ensuring the availability of a “seed bank” for the domination of a certain Chloroflexi species when its optimal growth conditions are met (Louca et al., 2018; Tully et al., 2018b).

Genes encoding for extradiol ring cleavage dioxygenases, monooxygenases, and laccases were found in nine of Chloroflexi SAGs. This suggests a potential for the degradation of aromatic compounds which are usually enriched in such WWTPs. In addition, the overuse of fungicides, insecticides, and pesticides in vineyards could have resulted in the accumulation of aromatic compounds in the wastewater (Cabras and Angioni, 2000; Esteve et al., 2009). Unfortunately, no complete degradation pathway could be inferred from Chloroflexi SAGs due to the incompleteness of the reconstructed genomes. It is hypothesized that Chloroflexi may be involved in the intermediate steps of aromatic compound degradation, including the ring cleavage, as it was previously demonstrated *via* genome analysis of SAR202 and *Caldilineae* from marine and sponge associated environments (Landry et al., 2017; Bayer et al., 2018). This type of “metabolic networking” has also often been observed in degradation of anthropogenic pollutants in which metabolites of one organism are channeled into the metabolic pathways of others (Pelz et al., 1999). This phenomenon has also been hypothesized as the “handoff” mode of metabolism occurring among rare bacteria belonging to the candidate phyla radiation in the subsurface environment (Anantharaman et al., 2016).

The ability of slow-growing bacteria to produce secondary metabolites such as terpene, type III polyketide synthases, non-ribosomal peptide synthases, thiopeptide, and bacteriocin, offers a defense mechanism against fast-growing microorganisms living in the same niches. Chloroflexi have previously been considered a potential source of secondary metabolites: e.g., isolates belonging to the class *Ktedonobacteria* and the genus *Herpetosiphon* exhibit broad antimicrobial activities against both Gram-positive and Gram-negative bacteria and harbor high numbers of secondary metabolite synthesis gene clusters (Livingstone et al., 2018; Zheng et al., 2019). Gene clusters for secondary metabolite synthesis were also found in genomes of uncultured *Caldilineae* and SAR202 associated with marine sponges (Bayer et al., 2018). Our finding of another potential secondary metabolite producer belonging to the class *Caldilineae* raises a hypothesis that this class might become an emerging candidate for production of biologically active compounds. With an increasing interest in searching for secondary metabolite producers *via* culture-independent techniques, targeted cell sorting can be revised by designing probes to capture more specific bacteria that have potential to produce such products.

## Conclusion

Our study provides a sensitive approach to capture extremely low-abundant albeit ecologically and biotechnologicially relevant microorganisms in the environment. This improved sensitivity currently still comes at the cost of reduced completeness in some genomes due to the biased nature of MDA. However, since sorted cells were effectively separated from their surrounding community in an originally intact state, the complete genome sequence should be potentially available, e.g., by future by improvements in methodology of FISH labeling, whole-genome amplification, or combination of enrichment sorting and direct sequencing of so-called mini-metagenomes, thereby circumventing the need for amplification all along. Nonetheless, the draft genomes obtained using our approach revealed novel phylogenies, metabolisms, and other physiological characteristics of rare members of the community that would have otherwise been overlooked by conventional metagenomics unless investing in substantially higher sequencing depth. This is especially exemplified by the identification of several potential genomic islands related to horizontal gene transfer in the SAGs but not the corresponding MAG, as such regions are unlikely to be correctly and unambiguously binned from metagenomes. Moreover, by placing a focus on specific organisms of interest, targeted cell sorting helps to reduce the costs of SCG to allow more microbial ecology laboratories access to this innovative methodology. Targeted cell sorting may even have potential as a novel isolation approach, specifically focusing on members of the “uncultivated majority.” Hence, this technique represents an essential complement to cultivation-based, metagenomics, and microbial community-focused research approaches for elucidating the genomic potential of novel taxa currently still hidden within the “microbial dark matter.”

## Materials and Methods

### Sample Collection and Preparation

Wastewater samples from the aerated lagoon (LEA) of the WWTP of the Establecimiento Juanicó winery (located in the village Juanicó in Canelones, Uruguay, latitude −34.6, longitude −56.25) were collected 20 cm below the water level. This treatment unit is the first in the WWTP and receives the crude effluent directly from the winery without previously passing through an equalization basin. The effluent composition, concentration, and pH vary greatly according to the winery operation. The volume of the aerated lagoon is 150 m^3^ and the sludge retention time (SRT) 3 days. Parameters of the aerated lagoon LEA at the different times of sampling are shown in Supplementary Table 5. The samples were vortexed at maximum speed for 3 min to release cells attracted loosely to the sediments. After 1 h, the sample was centrifuged at 2,500 rpm for 30 s to remove large particles (Rinke et al., 2014). The supernatant was filtered through a 30-μm polycarbonate membrane using gravity flow filtration force (CellTrics^®^ Filter, Partec, Muenster, Germany).

### Fluorescence *In situ* Hybridization (FISH)

Cells in the sample were hybridized with equal amounts of two probes labeled with Cyanine3 fluorochrome that target the phylum Chloroflexi: GNSB941 (5′-AAACCACACGCTCCGCT-3′) (Gich et al., 2001) and CFX1223 (5′-CCATTGTAGCGTGTGTGTMG-3′) (Björnsson et al., 2002). The hybridization protocol used in this study was modified from the protocol by Yilmaz et al. (2010) and Pernthaler et al. (2002). Cells were pelleted, washed twice with 1 × phosphate-buffered saline (PBS) to remove possible fluorescence molecules, and hybridized with the two probes, each at a final concentration of 15 ng/μL in 100 μL hybridization buffer containing 35% formamide at 46°C for 3 h in the dark. Labeled cells were washed twice with pre-warmed wash buffer at 48°C for 20 min each. Cells were then washed for the last time with ice-cold PBS buffer before being resuspended in 500 μL PBS buffer. The negative “no-probe” control was treated the same way as labeled samples except that no probes were added during the hybridization step. To test if the fluorescence signal of hybridized cells could be improved, cells were treated with increasing concentrations of ethanol (50, 80, and 98%) with 3-min incubation times (Haroon et al., 2013). Hybridized cells were visualized with Axiophot fluorescence microscopy (Carl Zeiss Microimaging GmbH). Labeled cells were stored in 5% glycerol at −80°C for sorting the next day with no loss of signal. In order to verify the specificity and sensitivity of labeling Chloroflexi, a mixed culture containing 1% *Sphaerobacter thermophilus* (DSM20745) and 99% *Escherichia coli* K12 (DSM498) was used. The hybridization procedure was carried out as described with the WWTP samples.

### Targeted Cell Sorting of Labeled Cells

Cell sorting of labeled cells was performed using a BD FACSAria III cell sorting system (BD, Heidelberg, Germany). A 488- and 561-nm laser was used as excitation source for light scattering and fluorescence, respectively. Hybridized cells were diluted by a factor of 5 in PBS, filtered through a 10-μm membrane (CellTrics^®^ Filter, Partec, Münster, Germany), and briefly sonicated in an Ultrasonic cleaner (VWR, Darmstadt, Germany) to break up cell clusters. Labeled cells were enriched by sorting into a 5-mL Falcon^®^ polypropylene tube (Corning, NY, United States) using purity sort mode. Cells from the enrichment sort were sorted into Hard-Shell^®^ 384-well plates (Bio-Rad Laboratories, Munich, Germany) using the single-cell mode of the FACS at a lower speed (50–100 cells/s). Cells were then sorted based on signal intensity of forward scattering and emitted fluorescence, compared to those of the no-probe control.

### Multiple Displacement Amplification (MDA)

Cells were lysed, and their genomic DNA was released during alkaline lysis at 65°C for 10 min. Genomic DNA was amplified with phi29 DNA polymerase at 30°C for 6 h using REPLI-g^®^ Single Cell Kit (Qiagen, Hilden, Germany) on a CFX384 Touch^TM^ Real-Time Detection System (Bio-Rad Laboratories, Munich, Germany). The whole-genome amplification was monitored in real time by detection of SYTO13^®^ (Life Technologies, CA, United States) fluorescence every 5 min. MDA reaction was then terminated at 65°C for 10 min. The cycle quantification (Cq) values and endpoint relative fluorescence units were used to determine the positive amplifications.

### 16S rRNA Gene Amplification and Screening

MDA products were diluted 1:20 and used as templates to amplify 16S rRNA genes with universal bacterial primer pairs: 926wF: 5′-AAACTYAAAKGAATTGRCGG-3′ and 1392R: 5′-ACGGGCGGTGTGTRC-3′ (Rinke et al., 2014). PCR products were cleaned up with DNA Clean and Concentrator-5 (Zymo Research, Freiburg, Germany) and subjected to Sanger sequencing. 16S rRNA gene sequences were blasted against the Silva SSU database (version 132, released in December 2017), and the identities of the corresponding single cells were performed using the web- based tool SINA Search and Classify on www.arb-silva.de (Pruesse et al., 2012).

### DNA Extraction for Metagenome Sequencing

DNA from the WWTP samples was extracted using a hexadecyltrimethylammonium bromide (CTAB)-based method with some modifications (Griffiths et al., 2000). 1.5 mL of the samples was centrifuged at maximum speed for 5 min to collect biomass. Pellets were then transferred into Lysing matrix E beads (MP Biomedicals, France). Five hundred μL 6% CTAB extraction buffer and 500 μL phenol:chloroform:isoamyl alcohol (25:24:1) were added into the extraction tube. Cells were lysed by vortexing at maximum speed on a Vortex Genie2 (Scientific Industries, NY, United States) for 3 min. Supernatant was extracted twice with phenol:chloroform:isoamyl alcohol (25:24:1) and twice with chloroform:isoamyl alcohol (24:1). The aqueous phase was transferred into a clean 1.5-mL tube. DNA was precipitated with 2.5 volume of 100% ethanol and 0.1 volume of 3 M sodium acetate (pH 5.2) and re-suspended in 50 μL PCR-grade water. Extracted DNA was cleaned up with the DNA Clean and Concentrator-5 kit (Zymo Research, Freiburg, Germany) as per the manufacturer’s instruction. A preliminary survey of microbial communities in WWTP samples was performed using pyrosequencing.

### Library Preparation for Metagenome and Single-Cell Genome Sequencing

Genomic DNA extracted from the WWTP samples and MDA products was quantified using the Qubit dsDNA HS Assay Kit (Thermo Fisher Scientific, OR, United States). Libraries were prepared using the NEBNext^®^ Ultra^TM^ DNA Library Prep Kit and NEBNext^®^ Ultra^TM^ II FS DNA Library Prep Kit (New England BioLabs, Frankfurt, Germany), respectively, following the manufacturer’s instruction. Five hundred nanogram of DNA was used as starting material. The quality of the DNA libraries was verified using the Agilent High Sensitivity DNA Kit on the Agilent 2100 Bioanalyzer instrument (Agilent Technologies, Germany). The libraries were then pooled and sequenced on Illumina systems using the paired-end approach and the highest available read length for each platform (150 bp for NovoSeq and NextSeq, 300 bp for MiSeq). Illumina platforms used to sequence SAGs are listed in Supplementary Table 6.

### Read Processing and Assembly

Quality trimming and adapter clipping were done using a three-step process, consisting of Trimmomatic v.0.36 (Bolger et al., 2014), bbduk v.35.69 (Bushnell, 2014), and cutadapt v.1.14 (Martin, 2011) using the following argument settings, respectively:

Trimmomatic: “ILLUMINACLIP: Trueseq3_PE.fa:2: 30:10 LEADING:3 TRAILING:3 SLIDINGWINDOW: 4:15 MINLEN:80”.
Bbduk: “-ktrim = r -mink = 11 -minlength = 45 -entropy = 0.25”.
Cutadapt: “-a AGATCGG$ -a CCGATCT$ -A AGATCGG$ -A CCGATCT$”.

Overlapping read pairs were identified and merged using FLASH v.1.2.11 (Magoč and Salzberg, 2011) with a minimum overlap of 16 bp, a maximum overlap of 100 bp, and a maximum mismatch fraction of 0.1. Residual contaminants of the Illumina PhiX control spike-in were removed using fastq_screen v.0.4.4 (Wingett and Andrews, 2018).

All datasets were assembled with SPAdes v.3.10.1 (Nurk et al., 2013), iterating through kmers 21-121 with a step size of 10 and using the “careful” argument. The “–sc” flag was used for all single-cell datasets, while the “–meta” flag was used for metagenome datasets. Winery metagenome samples obtained from different years (see Supplementary Table 5) were assembled individually and then subsequently merged using minimus2 (Sommer et al., 2007).

### Genome Assessment and Co-assembly

Genome completeness and purity was assessed using checkM (Parks et al., 2015). For taxonomic assignment, for additional purity assessments, and for decontamination purposes, a hierarchical least common ancestor (LCA) contig classification approach was performed as described by Pratscher et al. (2018), using preliminary assignments based on 16S rRNA, 23S rRNA, universal single-copy marker genes, and total protein sequences (Pratscher et al., 2018). Contigs with confident hierarchical taxon assignments that conflicted with the predominant taxon classification of the respective genome were removed as potential contaminations. The average nucleotide identity (ANI) approach implemented in pyani v.0.2.7 (Pritchard et al., 2016) was employed to identify groups of SAGs belonging to the same species, using a an identity cutoff of ≥99% identity and a coverage cutoff of 10%. SAGs of the same species were merged and reassembled into CAGs. SAGs with a genome coverage of less than 5% were omitted from analysis.

### Coverage Assessment and Binning

Metagenome coverage of all SAG, CAG, and merged metagenome contigs was obtained by mapping reads back to the assemblies using BamM v.1.7.3^3^. MAGs were obtained via metagenome binning by combining the results obtained from Maxbin v.2.2.6 (Wu et al., 2016), CONCOCT v.1.0.0 (Alneberg et al., 2014), and MetaBat v.2.12.1 (Kang et al., 2015) using DAS Tool v1.1.1 (Sieber et al., 2018). SAGs which, based on CheckM (Parks et al., 2015) evaluations and marker-gene phylogenies, potentially consisted of multiple co-sorted cells were separated into the respective potential component genomes by binning using Maxbin v.2.2.6 together with metagenome coverage information. After each binning and reassembly step, the completeness and purity of all bins and SAGs were reassessed using checkM (Parks et al., 2015) as well as the hierarchical contig classification procedure described in Pratscher et al. (2018).

### Phylogenetic Analysis of Chloroflexi CAGs, SAGs, and MAGs

Primary taxonomic assignments were inferred from the hierarchical contig classification results obtained during genome assessment (see section “Genome Assessment and Co-assembly” above). For comparison purposes, additional assignments were inferred using GTDB-TK (Chaumeil et al., 2019).

16S rRNA phylogenies were reconstructed using the Arb software package (Westram et al., 2011), which aligned 16S rRNA gene sequences amplified from Chloroflexi SAGs and CAGs, as well as selected reference Chloroflexi isolates. Streptomyces griseus was used as the outgroup. A phylogenetic tree was inferred using the neighbor joining algorithm with 1000 bootstrap permutations.

Proteinortho5 (v.5.16b) (Lechner et al., 2011) was used to detect groups of orthologous genes shared between reference genomes and CAGs, SAGs, and MAGs in our study with the following parameters: -identity = 25 -e = 1e-10 -cov = 60 -selfblast -singles. A gene-content-based genome clustering based on the presence or absence of genes from the bidirectional blast results of Proteinortho was implemented with a custom python script^4^ using the neighbor joining algorithm with 1000 bootstrap permutations. Streptomyces griseus was also used as an outgroup.

### Genome Analysis

Preliminary gene calling and annotations were inferred using different platforms including the Prokka pipeline (v1.12-beta), Rapid Annotations using Subsystem Technology (RAST) (Brettin et al., 2015), and Kyoto Encyclopedia of Genes and Genomes (KEGG) (Kanehisa and Goto, 2000). AntiSMASH (v4.1.0) (Blin et al., 2017) was used to identify putative secondary metabolite gene clusters.

### Pyrosequencing

DNA was extracted using the ZR Soil Microbe DNA MiniPrep^TM^ (Zymo Research, Irvine, CA, United States as described per the manufacturer’s instructions. DNA was dehydrated with 95% ethanol and submitted to the Institute for Agrobiotechnology Rosario (INDEAR, Rosario, Argentina) for 454-pyrosequencing and bioinformatic analysis (Roche Genome Sequencer FLX Titanium System). For sample LEA2013, the 16S rRNA genes were amplified with primers for the V4 region: 563f (5′-AYTGGGYDTAAAGNG-3′) and 802r (CAGGAAACAGCTATGACC) using a 10-bp barcode. For samples LEA2014 and LEA2015, the 16S rRNA genes were amplified with primers for the V3–V4 regions: 357F (5′-CACGACGTTGTAAAACGACCCTACGGGAGGCAGCAG-3′) /926R (5′-CAGGAAACAGCTATGACCCCGTCAATTCMTTTR AGT-3′) using a 10-bp barcode. Sequences were analyzed using the Quantitative Insights Into Microbial Ecology (QIIME) software (Caporaso et al., 2010).

Reads with length less than 200 bases, quality coefficient greater than 25, homopolymer size higher than six, and ambiguous bases were removed. Operational Taxonomic Units (OTU) were defined using the UClust algorithm based on 97% identity; OTUs that contained less than one sequence (singletons) were removed from the analysis. Reads were classified using the Classifier tool, from the Ribosomal Database Project^5^ with a cutoff of 50%.

## Data Availability Statement

The datasets generated for this study can be found under BioProject PRJNA589250 (SAGs) and BioProject PRJNA589250 (metagenome raw sequence reads).

## Author Contributions

HD and A-KK designed the study. HD and AC performed the experiments. HD and JV analyzed the data. A-KK and HD wrote the manuscript with assistance from JV and MS. A-KK and AC acquired the funding. AC provided the samples. All the authors read and approved the final manuscript.

## Conflict of Interest

The authors declare that the research was conducted in the absence of any commercial or financial relationships that could be construed as a potential conflict of interest.

## Footnotes

1 https://www.ncbi.nlm.nih.gov/

2 scgc.bigelow.org

3 http://github.com/ecogenomics/BamM

4 https://npm.pkg.github.com/jvollme/PO_2_GENECONTENT

5 https://rdp.cme.msu.edu/classifier/classifier.jsp
