# Supplementary material for: Targeted Cell Sorting Combined With Single Cell Genomics Captures Low Abundant Microbial Dark Matter With Higher Sensitivity Than Metagenomics
Source: Front Microbiol. 2020 Jul 22;11:1377. doi: 10.3389/fmicb.2020.01377 (PMC7387413; doi:10.3389/fmicb.2020.01377)
Supplement: Supplementary file 1 [file Data_Sheet_1.pdf]

Supplementary Data

Supplementary Tables

Supplementary Table 1. Overview of total bins obtained with the metagenomics binning approach.

Please refer to the separate file Supplementary_Table_1.xlsx

Supplementary Table 2. Overview of rejected potential Chloroflexi genomes contaminated by accidentally sorting multiple cells into one well.

Genomes	Completeness* (%)	Adjusted contamination*	Genome size (Mbp)	GC (%)	16S rRNA screening result
**Clx_SAG42**	30.17	3.45	0.89	37.7	*Anaerolineae*
**Clx_SAG43**	20.69	0	0.77	46.2	*Anaerolineae*
**Clx_SAG44**	3.45	0	0.12	50	*Caldilineae*
**Clx_SAG45**	51.78	3.45	1.96	50.2	*Anaerolineae*
**Clx_SAG46**	18.68	0.16	0.24	48.8	*Anaerolineae*

Supplementary Table 3. Gene clusters captured by Clx_SAGs8, 10 and 15 but not by the corresponding Clx_MAG1.

Please refer to the separate file Supplementary_Table_3.xlsx

Supplementary Table 4. Comparison of taxonomic assignments using the hierarchical contig classification and GTDB-TK approaches

Genome	Hierarchical contig classification	GTDB-TK classification
Clx_CAG1	Caldilineae	Chloroflexota;c__Anaerolineae;o__Caldilineales;f__Caldilineaceae;g__;s__
Clx_CAG2	Anaerolineae	Chloroflexota;c__Anaerolineae;o__Caldilineales;f__;g__;s__
Clx_CAG3	Caldilineae	Chloroflexota;c__Anaerolineae;o__Caldilineales;f__Caldilineaceae;g__;s__
Clx_SAG4	Anaerolineae	Chloroflexota;c__Anaerolineae;o__;f__;g__;s__
Clx_SAG5	Caldilineae	Chloroflexota;c__Anaerolineae;o__Caldilineales;f__Caldilineaceae;g__;s__
Clx_SAG6	Anaerolineae	Chloroflexota;c__Anaerolineae;o__Anaerolineales;f__Anaerolineaceae;g__T78;s__
Clx_SAG7	Unclassified Chloroflexi	Patescibacteria;c__Dojkabacteria;o__SC72;f__SC72;g__UBA12078;s__
Clx_SAG8	Ardenticatenia	Chloroflexota;c__Anaerolineae;o__Promineofilales;f__Promineofilaceae;g__Promineofilum;s__
Clx_SAG9	Caldilineae	Chloroflexota;c__Anaerolineae;o__Caldilineales;f__Caldilineaceae;g__;s__
Clx_SAG10	Ardenticatenia	Chloroflexota;c__Anaerolineae;o__Promineofilales;f__Promineofilaceae;g__Promineofilum;s__
Clx_SAG11	Caldilineae	Chloroflexota;c__Anaerolineae;o__Caldilineales;f__Caldilineaceae;g__;s__
Clx_SAG12	Anaerolineae	Chloroflexota;c__Anaerolineae;o__Anaerolineales;f__Anaerolineaceae;g__UBA6107;s
Clx_SAG13	Anaerolineae	Chloroflexota;c__Anaerolineae;o__Anaerolineales;f__Anaerolineaceae;g__;s__
Clx_SAG14	Anaerolineae	Chloroflexota;c__Anaerolineae;o__Anaerolineales;f__Anaerolineaceae;g__UBA700;s__
Clx_SAG15	Ardenticatenia	Chloroflexota;c__Anaerolineae;o__Promineofilales;f__Promineofilaceae;g__Promineofilum;s__
Clx_SAG16	Cand.Thermofonsia	Chloroflexota;c__Anaerolineae;o__SBR1031;f__A4b;g__ZC4RG36;s__
Clx_SAG17	Anaerolineae	Chloroflexota;c__Anaerolineae;o__Anaerolineales;f__Anaerolineaceae;g__UBA6107;s
Clx_SAG18	Chloroflexia	Chloroflexota;c__UBA6077;o__UBA6077;f__UBA6077;g__UBA6077;s__
Clx_SAG19	Caldilineae	-
Clx_MAG1	Ardenticatenia	Chloroflexota;c__Anaerolineae;o__Promineofilales;f__Promineofilaceae;g__Promineofilum;s__
Clx_MAG2	Thermomicrobia	Chloroflexota;c__Chloroflexia;o__Thermomicrobiales;f__UBA6265;g__UBA6265;s__
Clx_MAG3	Anaerolineae	Chloroflexota;c__Anaerolineae;o__Anaerolineales;f__Anaerolineaceae;g__49-20;s__
Clx_MAG4	Ardenticatenia	Chloroflexota;c__Anaerolineae;o__Promineofilales;f__Promineofilaceae;g__Promineofilum;s__

Supplementary Table 5. Parameters of the aerated lagoon LEA in the Establecimiento Juanico winery waste water treatment plant at different sampling times over a three-year period.

Sample	Collection date	SS60 (cm^3/L)	COD (mgO_2/L)	pH	No. Sequence Reads	NCBI SRA Accession
**LEA2013**	Nov. 2013	300	4,350	6.5	22 million	SRR10961543
**LEA2014**	Jan. 2014	150	NA	7.8	20 million	SRR10961542
**LEA2015**	Oct. 2015	800	NA	6.4	176 million	SRR10961541

SS: Settleable Solids – a measurement of the settlement properties of the sludge, measured with an Imhoff cone for 60 minutes.
COD: Chemical Oxygen Demand
NA: not analyzed

Supplementary Table 6. Sequencing of single cells was done on Illumina MiSeq® or NovaSeq® platforms, while all metagenomes were sequenced on a NextSeq® system. The paired end approach and highest available read lengths were used for all sequencing runs (150 bp for NextSeq and NovaSeq, 300 bp for MiSeq)

Dataset	Illumina Platform
**Clx_CAG1**	MiSeq & NovaSeq
**Clx_CAG2**	MiSeq
**Clx_CAG3**	MiSeq & NovaSeq
**Clx_SAG4**	NovaSeq
**Clx_SAG5**	NovaSeq
**Clx_SAG6**	NovaSeq
**Clx_SAG7**	NovaSeq
**Clx_SAG8**	MiSeq
**Clx_SAG9**	NovaSeq
**Clx_SAG10**	NovaSeq
**Clx_SAG11**	NovaSeq
**Clx_SAG12**	MiSeq
**Clx_SAG13**	NovaSeq
**Clx_SAG14**	NovaSeq
**Clx_SAG15**	MiSeq
**Clx_SAG16**	NovaSeq
**Clx_SAG17**	NovaSeq
**Clx_SAG18**	NovaSeq
**Clx_SAG19**	NovaSeq

Supplementary Table 7: Read coverage of CAGs, SAGs and MAGs across Uruguay wastewater metagenome samples

	LEA2013	LEA2014	LEA2015
Clx_CAG1	0,00	0,00	0,00
Clx_CAG2	0,21	0,02	3,54
Clx_CAG3	0,00	0,00	0,72
Clx_SAG4	0,00	0,00	0,07
Clx_SAG5	0,00	0,00	0,00
Clx_SAG6	0,14	0,28	4,38
Clx_SAG7	0,00	0,00	0,05
Clx_SAG8	25,20	1,29	11,03
Clx_SAG9	0,29	0,00	0,09
Clx_SAG10	25,58	1,45	12,25
Clx_SAG11	0,04	0,00	0,09
Clx_SAG12	0,00	0,00	8,06
Clx_SAG13	0,00	0,00	0,00
Clx_SAG14	0,00	0,00	0,00
Clx_SAG15	25,39	1,57	13,58
Clx_SAG16	0,00	0,00	0,04
Clx_SAG17	0,00	0,00	0,26
Clx_SAG18	0,00	0,00	0,00
Clx_SAG19	0,00	0,00	0,00
Clx_MAG1	24,37	1,44	12,19
Clx_MAG2	0,00	0,00	4,27
Clx_MAG3	0,00	0,00	5,83
Clx_MAG4	5,28	0,18	1,95

Supplementary Table 8. Comparison table listing checkM metrics of CAGs, SAGs and MAGs based on Chloroflexi-specific marker gene sets

Genome	Completeness (%)*	CheckM contamination Stats [Cont./SH/Adj-Cont.] (%)*	Size (Mbp)	GC (%)
**CAGs and SAGs**				
**Clx_CAG1**	84.26	9.12/2.63/**8.89**	6.97	53.4
**Clx_CAG2**	61.01	1.95/16.67/**1.62**	3.41	62.5
**Clx_CAG3**	33.46	3.09/0/**3.09**	1.91	58.9
**Clx_SAG4**	49.43	1.34/0/**1.34**	2.12	61.3
**Clx_SAG5**	42.8	1.68/0/**1.68**	3.37	52.9
**Clx_SAG6**	52.76	2.82/0/**2.82**	1.73	49.1
**Clx_SAG7**	36.03	1.86/50/**0.93**	0.63	36.1
**Clx_SAG8**	14.36	0.02/0/**0.02**	0.72	50.6
**Clx_SAG9**	22.14	0/0/**0**	1.42	63.0
**Clx_SAG10**	18.58	0/0/**0**	1.10	57.6
**Clx_SAG11**	9.76	0/0/**0**	0.51	60.6
**Clx_SAG12**	24.3	0.34/0/**0.34**	0.72	50.6
**Clx_SAG13**	6.83	0.02/0/**0.02**	0.88	47.3
**Clx_SAG14**	22.93	0/0/**0**	0.58	45.4
**Clx_SAG15**	13.65	1.34/50/**0.67**	1.18	61.9
**Clx_SAG16**	17.99	0.17/0/**0.17**	1.72	59.0
**Clx_SAG17**	19.66	0/0/**0**	0.87	45.4
**Clx_SAG18**	15.94	0.67/0/**0.67**	0.54	60.0
**Clx_SAG19**	14.33	0.67/0/**0.67**	0.12	46.6
**MAGs**				
**Clx_MAG1**	93.2	9,48/73,6/**2,5**	5.32	61.4
**Clx_MAG2**	65.88	0/0/**0**	1.69	60.5
**Clx_MAG3**	65.51	3,38/50/**1,69**	1.64	55.7
**Clx_MAG4**	50.21	2,73/28,5/**1,95**	3.09	64.4

* Completeness and contamination estimations were based on CheckM (Parks et al., 2015) results using Chloroflexi-specific marker sets. The CheckM "contamination" stats are given in the form of three values: "Cont."= original CheckM contamination estimate (Cont.), based on the number of duplicate markers; "SH"= "strain heterogeneity" indicating the fraction of duplicate markers with almost complete sequence identity not reflecting cross species contamination; "Adj.-Cont." = "adjusted contamination" giving the fraction if duplicate marker genes with distinct sequences

Supplementary Figures

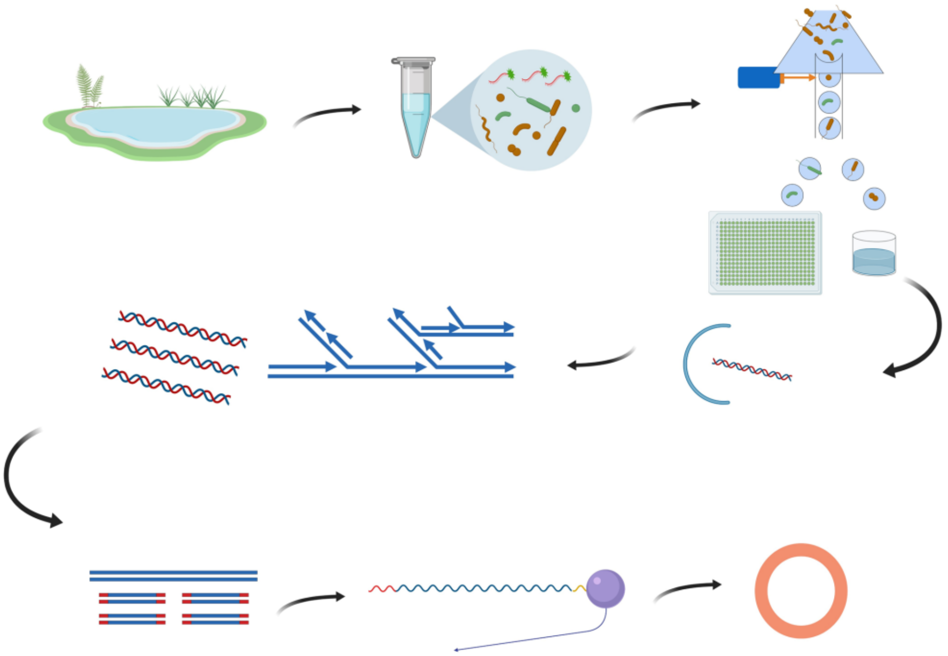

Supplementary Figure 1. Workflow of targeted cell sorting combined with single cell genomics. Samples from the environment were pretreated to remove large particles, cells of interest were labeled with phylogenetically specific probes using in-solution fixation-free fluorescence *in situ* hybridization (FISH). Labeled cells were sorted using Fluorescence activated cell sorter into 384 well plates. Prior to sorting into 384 well plates, enrichment sort was performed to enrich for cells of interest. Sorted cells were lysed to release their genomic DNAs which were then amplified via multiple displacement amplification to generate sufficient quantity of DNA for subsequent steps. The resulting single amplified genomes (SAGs) were subjected to PCR using broad bacterial primers to amplify 16S rRNA gene fragments, followed by Sanger sequencing to identify their phylogenetic affiliations. SAGs of the cells of interest were used to prepare DNA libraries for high throughput sequencing on the Illumina platform. Bioinformatics was used to reconstruct genomes of the targeted single cells, metabolisms and physiological properties were inferred from the reconstructed genomes. The figure was made with BioRender.com and is based on a previous description by Kaster et al. (2014).

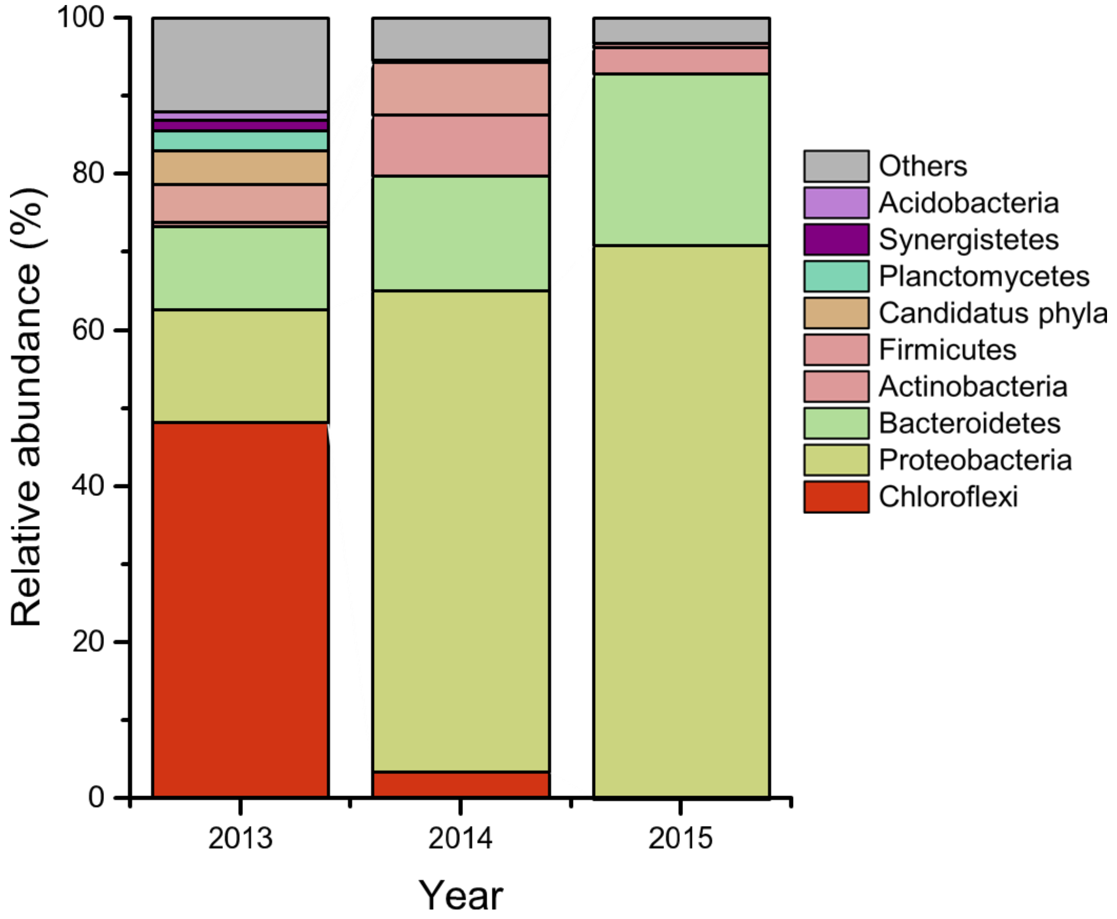

Supplementary Figure 2. Taxonomic composition and diversity of LEA samples based on pyrosequencing. Samples were collected in three consecutive years and microbial diversity was analyzed by pyrosequencing of the V3 – V5 regions of the 16S rRNA gene.

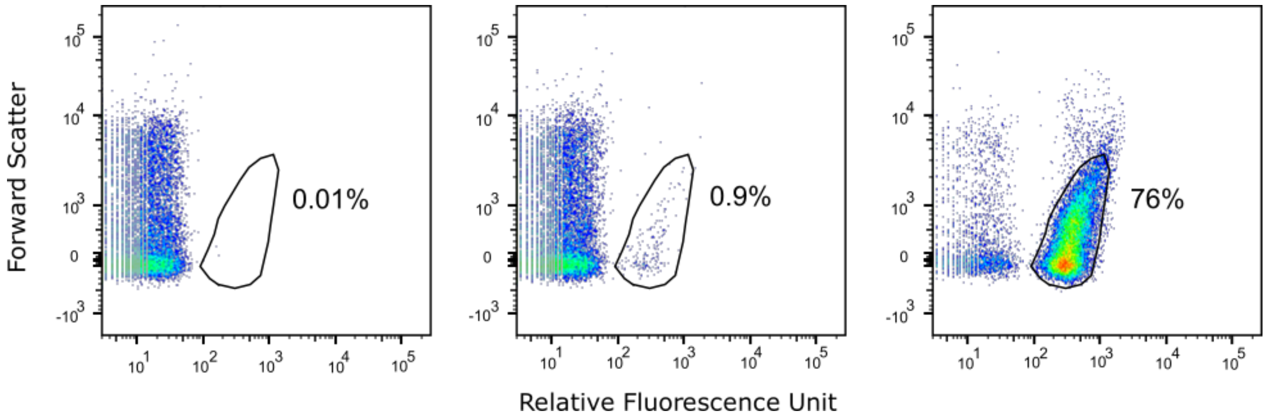

Supplementary Figure 3. Flow cytometric analysis of FISH-labeled *Sphaerobacter thermophilus* cells in a mixed culture with 99% *E. coli*. (A) Negative control without probes. (B) Gated population after the first sort. (C) Gated population after the second sort. Cells in the mixed culture were labeled with two Chloroflexi-specific probes (CFX1223 and GNSB941) in an in-solution fixation-free fluorescence *in situ* hybridization. A total of 20,000 events were recorded. Numbers indicate the percentage of the gated population in the total event recorded.

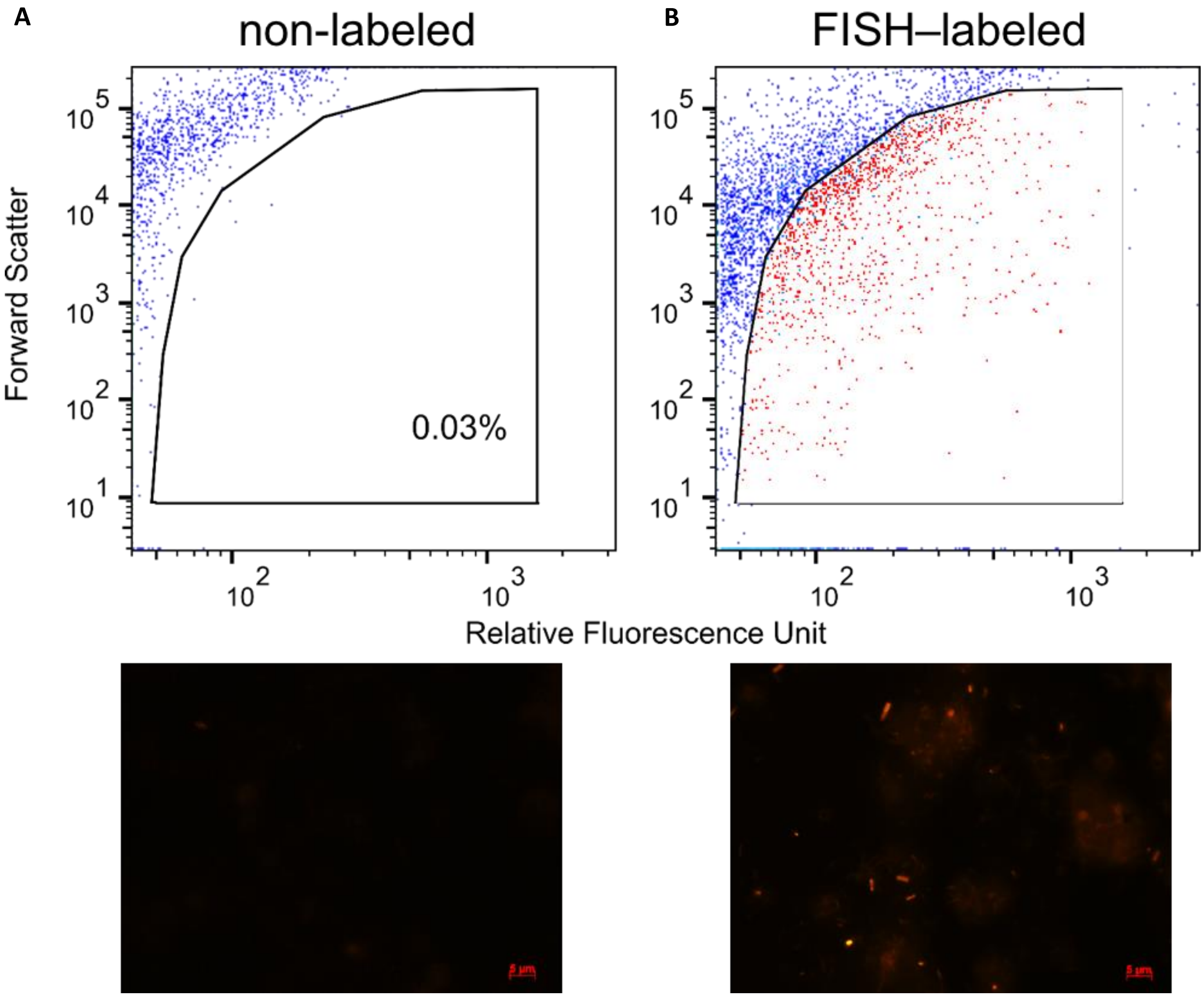

Supplementary Figure 4. Epifluorescence analysis of labeled cells via fluorescence *in situ* hybridization (FISH) in wastewater sample LEA2015. (A) Negative control without probes. (B) FISH-labeled sample. Cells were hybridized with two Chloroflexi-specific probes (CFX1223 and GNSB941) in an in-solution fixation-free FISH. A total of 20,000 events was recorded. Population of labelled cells in the closed area was sorted.

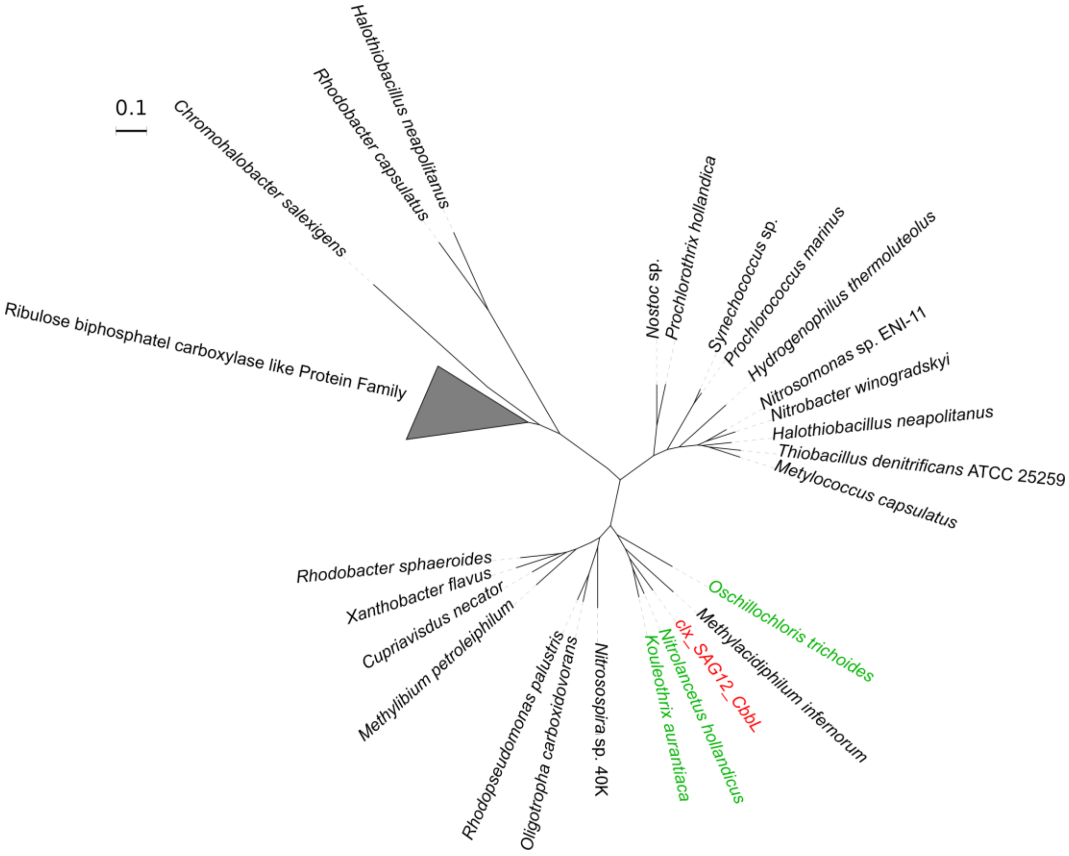

Supplementary Figure 5. Phylogeny of ribulose bisphosphate carboxylase large subunit (CbbL). CbbL from known autotrophic Chloroflexi isolates and the single cell in this study are presented in green and red, respectively. Scale bar indicates a 10% sequence divergence.

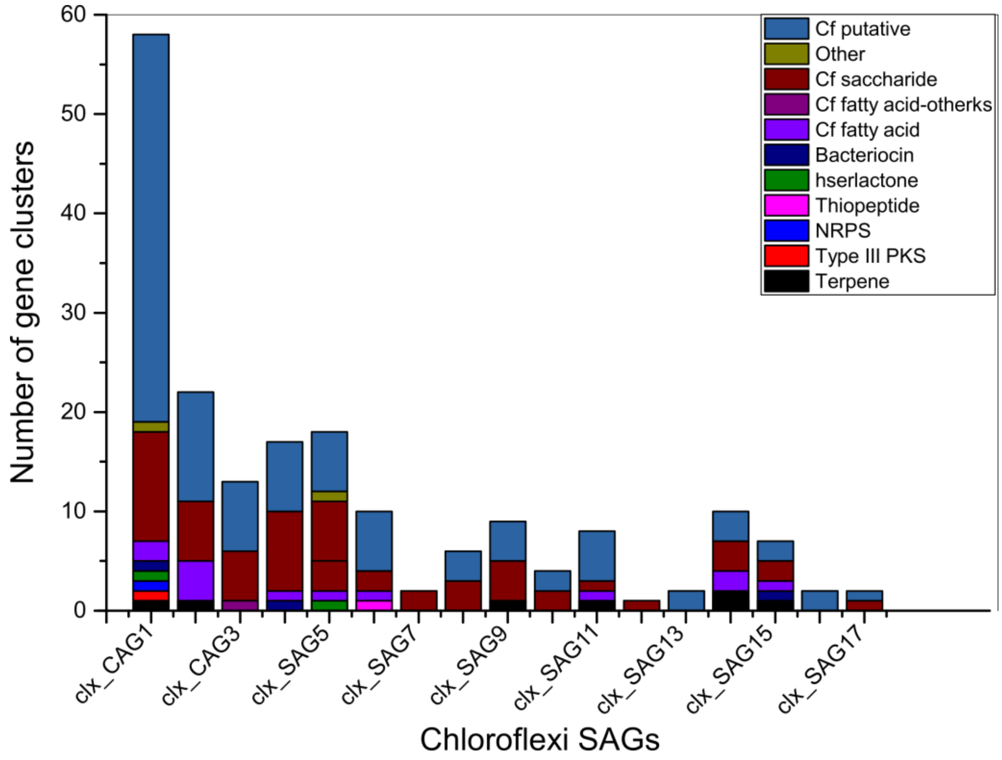

Supplementary Figure 6. Secondary metabolite synthesis gene clusters of Chloroflexi single cells identified by the tool antiSMASH (Blin et al., 2017).

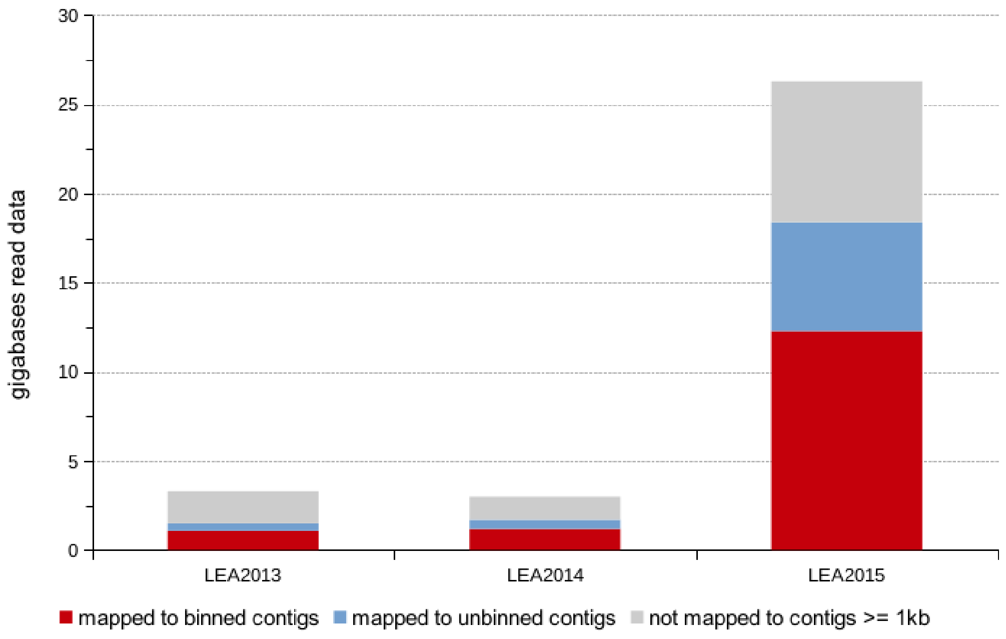

Supplementary Figure 7. Fraction of metagenomic read data in gigabases contributing to the metagenomic assembly and binning.

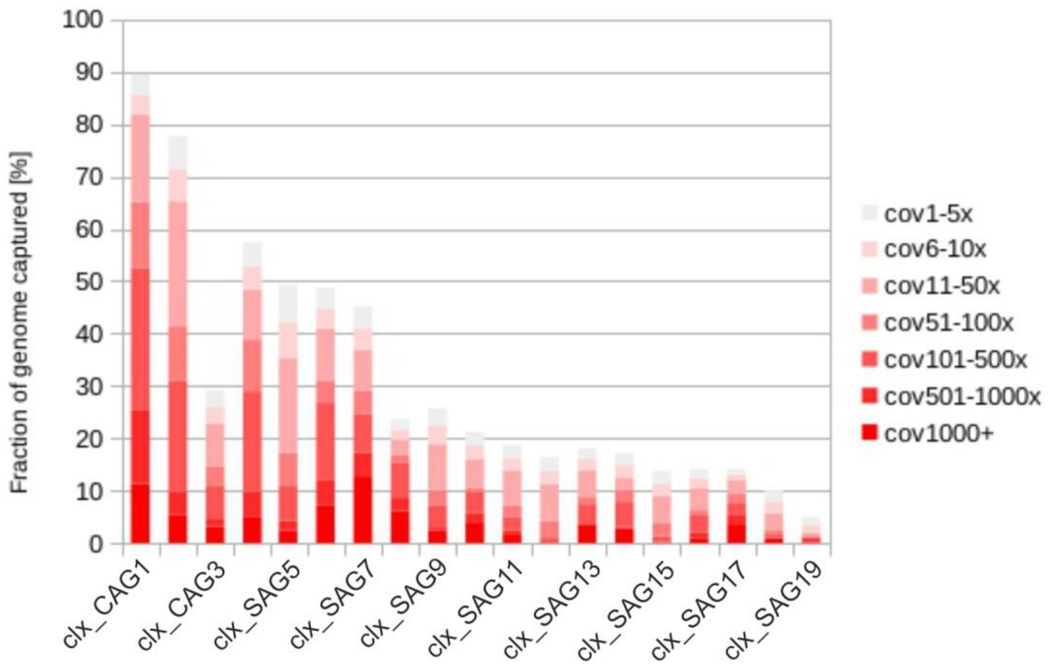

Supplementary Figure 8: Coverage distribution across the SAG assemblies. Total bar heights indicate the estimated genome completeness for each bin derived by checkM analyses. The proportion of each SAG displaying different degrees of coverage are indicated by a red color gradient.

References

Blin, K., Wolf, T., Chevrette, M. G., Lu, X., Schwalen, C. J., Kautsar, S. A., et al. (2017). antiSMASH 4.0—improvements in chemistry prediction and gene cluster boundary identification. *Nucleic Acids Res.* 45, W36–W41. doi:10.1093/nar/gkx319.

Kaster, A.-K., Mayer-Blackwell, K., Pasarelli, B., and Spormann, A. M. (2014). Single cell genomic study of Dehalococcoidetes species from deep-sea sediments of the Peruvian Margin. *ISME J.* 8, 1831–42. doi:10.1038/ismej.2014.24.

Parks, D. H., Imelfort, M., Skennerton, C. T., Hugenholtz, P., and Tyson, G. W. (2015). CheckM: assessing the quality of microbial genomes recovered from isolates, single cells, and metagenomes. *Genome Res.* 25, 1043–55. doi:10.1101/gr.186072.114.
